# Editorial Perspective: Cabin fever – the impact of lockdown on children and young people

**DOI:** 10.1111/camh.12458

**Published:** 2021-03-22

**Authors:** Paul Crawford

**Affiliations:** ^1^ Centre for Social Futures Institute of Mental Health University of Nottingham Innovation Park Nottingham UK

## Abstract

This article debates the impact of the pandemic lockdown on the mental health of children and young people. It proposes that children and young people have been subject to the kind of psychological distress that has featured as the folk syndrome, cabin fever. Drawing on the evidence about the impact of prolonged confinement and isolation on mental health, not least in penal and spaceflight contexts, the article points to a long tail of mental health challenges for children and young people through and in the wake of the pandemic. Finally, the article summarizes some of the antidotes for cabin fever and new, exciting, creative digital interventions that may assist upstream mental health literacy and complement and support the work of child and adolescent mental health services.

The COVID‐19 pandemic and prolonged confinement and isolation during lockdown measures have had a deleterious impact on the mental health of children and young people. The social contexts for children and young people during this last year have been markedly different to what they will have experienced before. Indeed, they have been living through ‘the greatest confinement in history’ (Crawford & Crawford, 2021). Children and young people have been subject to disrupted education at school, college, and university, as well as hampered transition into training or the workforce for the first time. In addition to these contexts, many children and young people will have experienced strained relationships within their family units as parents anticipate or are subject to insecure futures due to unemployment, inability to pay for accommodation, or other major changes that have occurred such as combining working from home with involuntary homeschooling. Importantly, these challenging social changes have compounded the psychological challenges and distress known as cabin fever.

In *Cabin Fever: Surviving Lockdown in the Coronavirus Pandemic* (Emerald, 2021), we examine the mental health fallout of voluntary and involuntary sequestering in people’s homes, alone or with family members, friends, or even strangers, to halt the spread of the virus. The pandemic lockdown brought heightened concern from many leading bodies, not least the World Health Organization, about the mental health challenges of this radical social change. As Holmes et al. (2020) note, social isolation and loneliness are ‘strongly associated with anxiety, depression, self‐harm, and suicide attempts across the lifespan’. In the book, we examine the development of the notion of ‘cabin fever’ from prolonged confinement and isolation.

Cabin fever is not a medically defined condition but a ‘folk syndrome’ commonly understood to refer to a combination of anxiety, lassitude, irritability, moodiness, boredom, depression, or feeling of dissatisfaction in response to confinement, bad weather, routine, isolation, or lack of stimulation. A person subject to cabin fever may suffer from sleeplessness (insomnia) or sleepfulness (hypersomnia). They may even develop paranoia and difficulty in rational decision‐making. At its extreme, people may feel compelled to escape their spatial restrictions or limited routines, regardless of external conditions or the cost to themselves or others. Cabin fever may also lead to self‐ and other‐directed violence, including suicide.

Like those confined in prison, especially solitary confinement, who go ‘stir crazy’, or those wintering through in remote regions, or even experiencing long voyages at sea or in space (on indeed, in Arctic or other analogue stations preparing for the trip to Mars one day), many children and young people are experiencing challenging if not disturbing confinement and isolation.

While scientists in analogue stations or in space missions successfully endure long periods in confinement and isolation, this is not always without psychological deterioration despite the mitigation of having a trained crew, explicit goals, purpose, and schedule. In contrast, many children and young people during this last year have been subject to long periods indoors or with limited social engagement without the trained‐in resilience, scheduling, and purpose of astronauts. Many will be doing their space travel on the ground without the benefits of a supportive crew, mission, etc. Their own families may be disorientated, chaotic, or dysfunctional in the lockdown experience.

In her book, *Solitary Confinement: Social Death and its Afterlives* (2013), Lisa Guenther underlines the damage of social confinement in reference to the US penal system and how this can have a profound and deleterious impact on a person’s psychological, social, and sense of identity. She writes, ‘Deprived of meaningful human interaction, otherwise healthy prisoners become unhinged. They see things that do not exist, and they fail to see things that do’ (2013, p. xi). While children and young people have generally not been subject to solitary confinement, we should not dismiss the symptoms among prisoners of ‘anxiety, fatigue, confusion, paranoia, depression, hallucinations, headaches and uncontrollable trembling’ (2013, p. xii). Guenther goes on to outline how such confinement brings a ‘social death’, and inmates have to learn ways to adjust to the expanse of ‘dead time’ that lies ahead. The deleterious impact of solitary confinement in the penal setting is to some extent analogous to children and young people living for prolonged periods during lockdown at home – which may be more or less commodious and resourced. It is clearly not the same but bears our consideration, especially in relation to coping with monotony, boredom, or ‘dead time’.

We already know that mental health deteriorates with the social isolation of quarantine (Brooks et al., 2020) and reports since the pandemic began have alerted us to the impact of prolonged isolation on the mental health of children and young people in terms of increased depression, anxiety, and loneliness (Mind, 2020; Public Health England, 2020). Indeed, NHS Digital (2020) found that mental health problems now affected 1 in 6 children and young people compared with 1 in 9 in 2017. The Office for National Statistics (ONS, 2020) also discovered more than half of all students at university (57%) reported a worsening in their mental health and well‐being since the beginning of the autumn term in September 2020. In terms of the latter, we have seen reports in the press about how young people at university experienced prisonlike, quarantine conditions and felt ‘caged’ within their halls of residence. These and other social dislocations have affected this particular age group, robbing them of their expected social freedom of higher education and quite possibly traumatizing them. The monotony and threat of lockdown life that has replaced this longed‐for rite of passage have been closer to the kind of experiences afforded prison inmates or those who suffered quarantine on cruise ships.

Fortunately, we know that there are several potential antidotes to cabin fever: accessing outdoor space, not least nature; acceptance of the ‘new normal’; social connectedness (largely achieved digitally during the pandemic); working as a ‘crew’, setting goals and purpose to each day; conceiving home during lockdown as a sanctuary rather than a prison; looking after the body (nutrition, hydration, sleep, exercise); and engaging with or experiencing creativity through the arts, crafts, and humanities (Crawford & Crawford, 2021).

Practitioners working to support the mental health of children and young people will have much to do now and over the coming months and even years because the mental health tail of the pandemic will be longer than the physical assault. For those children and young people who have lost parents, grandparents, or other close family members to COVID‐19 or in response to it, their bereavement will carry forward. For those who have lost chances or educational momentum through this period, their confidence and hope in the future may remain low for some time and require boosting. Many more will have been subject to or witnessed sexual and domestic violence, something that has increased during lockdown. Others will require help with depression, anxiety, and self‐harm. We will all need to be creative to meet these challenges upstream and downstream.

One example of a creative, upstream campaign to support young people’s mental health is launched in February this year and is called *What’s Up With Everyone?* I led this project with the Academy Award‐winning Aardman (*Wallace & Gromit*, *Shaun the Sheep,* etc.) alongside diverse academic, clinical, and charitable partners to provide accessible, engaging mental health literacy resources. Aimed at 17‐ to 24‐year‐olds but also accessible to a younger age range, it comprises five animated stories dealing with life challenges that young people told us they found tricky just now: loneliness and isolation, perfectionism, competitiveness, social media, and independence. We hope that these films and additional mental health information available on the platform www.whatsupwitheveryone.com will help our young people a little at this difficult time. Given the pressures that will face child and adolescent mental health services over the coming months and years, these kinds of creative public health initiatives may prove a welcome shot in the arm.

## Ethical information

No ethical approval was required for this article.
